# Healing online? Social anxiety and emotion regulation in pandemic experience

**DOI:** 10.1007/s11097-023-09886-2

**Published:** 2023-02-27

**Authors:** Anna Bortolan

**Affiliations:** Department of Politics, Philosophy and International Relations, Swansea University, Singleton Park, Swansea,, SA2 8PP UK

**Keywords:** Internet, Mental health, Social anxiety, Embodiment, Pandemic, Emotion

## Abstract

During the pandemic of Covid-19, internet-based communication became for many the primary, or only, means of interaction with others, and it has been argued that this had a host of negative effects on emotional and mental health. However, some people with a lived experience of mental ill-health also perceived improvements to their wellbeing during the period in which social activities were moved online.

In this paper, I explore the possibility that some of these improvements are due to the partial “disembodiment” of emotions facilitated by internet-mediated interaction. In particular, I consider the phenomenology of social anxiety and how it may be impacted upon by encountering others primarily through the medium of internet-enabled technology.

I will start by reconstructing a phenomenological account of social anxiety to which disruptions of bodily experience are central. I will then move to consider how the experiential dynamics that are particularly prominent in social anxiety can be weakened when communicating with others via video calls, instant messages, and social media more broadly. I will suggest that this is the case due to the diminished visibility of the body online, and the higher degree of control and agency over one’s experience that can be exercised in this context.

Finally, I will argue that the weakening of social anxiety through internet-mediated contact exemplifies some of the processes which are key to emotion regulation more widely, thus suggesting that communication and interaction online could have a positive effect on a wider range of affective disturbances.

## Covid-19 and phenomenology: the role of embodiment

A growing body of diverse philosophical work has appeared in response to the pandemic of Coronavirus disease (e.g. Amoretti & Lalumera, 2021; Bramble, 2020; Depraz, 2021; Dolezal & Lucas, 2022). At this time, in addition to the urgent need to answer questions concerning how individuals and institutions should respond to an unprecedented public health crisis, the importance of understanding how people’s experience has been affected has also emerged clearly. To this extent, the methodological and theoretical frameworks of philosophical phenomenology have proved extremely useful, being drawn upon in a range of studies which have explored how the sense of self, others and the world have been transformed by the pandemic and the measures that have been undertaken to respond to it (e.g. Carel, 2020; Carel et al., 2020; Froese et al., 2021; Petherbridge, 2022).

In this context, significant attention has been devoted to the experiential changes that have been brought about by the implementation of lockdowns and social distancing, especially in the early stages of the pandemic. Work in this area has shed light on the dramatic ways in which our everyday life has been altered, emphasising the centrality of the reduction of face-to-face interactions in these dynamics.

Part and parcel of these processes has been the migration to online spaces of significant portions of our personal and professional lives. During the pandemic, many people have indeed not only been working, but also socialising, relaxing, learning, and mobilising via a range of internet-mediated technologies.

While this has made it possible to maintain a level of continuity in our lives despite facing critical disruptions, for a number of people video and conference calls, instant messages, and exchanges on social media have felt as an inadequate replacement of face-to-face interaction. Phenomenological research on pandemic experience suggests that this may be due, at least in part, to the very different role played by the body in online communication. For example, in her discussion of the lived experience of lockdown, Havi Carel offers the following observations:

When I recently asked my 7-year-old son if he wanted to talk to a friend online, he replied angrily: “No! What’s the point if I can’t touch him!?” His exasperation expresses a basic aspect of human social life: it is embodied. Talking to someone online is a poor replacement for embodied engagement with others. When we are with other people we hug and huddle, we look into each other’s face as we share a joke, we walk together, eat together, cuddle on the sofa as we share a film or talk. For children, this embodied dimension of social exchange is even more dominant[...]. (Carel, 2020: 12)

In both classical and contemporary phenomenology, attention has often been drawn to the centrality of bodily expressions, habits, and practices in enabling and sustaining intersubjective understanding, sociality, and affectivity (cf. Zahavi, 2001; Fuchs & Koch, 2014). From this perspective, for example, empathy has been characterised as rooted in the ability to directly perceive other people’s mental states through their facial expressions, gestures, and movements, and interpersonal relatedness has been seen as dependent on the capacity to resonate with and attune to them through a range of bodily capacities, routines, and affects (cf. Scheler, 1954; Gallagher & Zahavi, 2012: 208 ff.). As such, within the phenomenological tradition, face-to-face, embodied interaction has been viewed as a natural or typical way of being with others, and its disruption during the pandemic has been interpreted as a pathological phenomenon (Carel, 2020: 17).

Some of the authors who have explored from a phenomenological perspective life at the time of Covid-19 have also recognised that the pandemic did not involve only negative experiences, and in fact have acknowledged that, in some cases, positive personal (and, potentially, societal) transformations have been triggered by the upheavals put in motion by Coronavirus. However, when it comes to the modalities of interpersonal and social relatedness that have been fostered by responses to the pandemic, the assessment remains quite pessimistic. “Practically and socially,” Carel & Kidd (2020: § 1) for example claim, “our lives have transformed, often in ways that are clearly poor substitutes for what came before [...]”.

I share the view that the pandemic has generated some profoundly disruptive, painful, and traumatic experiences and that phenomenology can be a very fruitful approach when trying to understand these phenomena. I also agree that one of the main dimensions to be considered in this context is embodiment, and how this has been affected by the reduction of face-to-face contact and the exponential rise of internet-mediated sociality.^1^

Nevertheless, existing philosophical, and in particular, phenomenological research on the topic has not yet given enough consideration to a range of pandemic experiences that have a more positive character, and which seem, at least in part, to have been made possible by the degree of “dis-embodiment”^2^ facilitated by social distancing, and the ways in which we have been relating to each other in, and through, cyberspace.

A range of first-person reports and recent research indeed suggest that some people with an experience of mental ill-health have noticed improvements to their wellbeing during the pandemic (cf. e.g. Jarral, 2020; Khazan, 2021; Thygesen et al., 2021), and some of these improvements appear to be connected to the possibility of interacting remotely rather than in person. This corresponds to my own personal experience as an anxiety sufferer, and is one of the reasons I became interested in the investigation of this topic. The pandemic undoubtedly multiplied the range of situations, events, or thoughts that could trigger my anxiety, as an expanded set of health-related concerns became very prominent. However, as the pandemic progressed, some of the changes in communication and interaction practices brought about by social distancing appeared to have a mitigating effect on a range of symptoms associated with my being persistently, and sometimes very acutely, anxious.

In the rest of this paper, I will explore the possibility that some of the reported positive effects of pandemic experience are due to the partial disembodiment that social relations have undergone at this time. In particular, my focus will be on the experience of social anxiety and how this may be impacted upon by the shift from face-to-face to internet-mediated modes of relatedness.

I will start by outlining a phenomenological account of social anxiety disorder (SAD) to which disruptions of bodily experience are central. Drawing on Tanaka’s (2021) and my own previous work (Bortolan, 2022), I will highlight how those who suffer from social anxiety tend to experience a heightening of reflective self-consciousness, an integral aspect of which is an increased awareness of one’s body as an object of attention and observation for others.

I will then move to consider how the experiential dynamics that are particularly prominent in SAD (but which are also common in various instances of experience that may not meet the criteria for a SAD diagnosis) can be weakened in internet-mediated communication and interaction. More specifically, I will suggest that this is the case due to the diminished visibility of the body online, and the higher degree of control and agency over one’s experience that can be exercised in cyberspace.

Finally, I will proceed to explore the implications of these insights for our understanding of emotions on the internet more broadly. I will suggest that the weakening of social anxiety through internet-mediated contact exemplifies some of the dynamics which are key to emotion regulation, thus suggesting that communication and interaction online could have a positive effect on a wider range of affective experiences.

## The experience of the body and social anxiety

Social anxiety disorder (SAD)^3^ is one of the anxiety disorders listed in the text revision of the fifth edition of the *Diagnostic and Statistical Manual of Mental Disorders* (DSM-5-TR), which describes this experience as “a marked, or intense, fear or anxiety of social situations in which the individual may be scrutinized by others”, and such fear or anxiety typically results in social situations being endured with distress or avoided (APA, 2022: 229–230).

Cognitive-behavioural models of the disorder suggest that social anxiety occurs in relation to the presence of an audience that has the potential to evaluate the self, and stems from distorted judgements concerning the evaluative standards of such audience, and the likelihood that one will be negatively evaluated by them (Rapee & Heimberg, 1997; Wong et al., 2014). The relevant evaluations can concern a range of features of the self, and as such, while the concern with negative appraisal is a key aspect of social anxiety, it is possible to be socially anxious in a variety of ways. As outlined by the DSM (APA, 2022: 230), for instance, the person who is socially anxious can indeed be worried that they “will be judged as anxious, weak, crazy, stupid, boring, intimidating, dirty, or unlikable”.

However, physical appearances and expressions are often the focus of, or play a central role in, these perceived assessments. Negative bodily self-images are reported as a feature of social anxiety episodes (Hackmann et al., 1998), and the body frequently becomes a cause of concern because of the ways in which it can manifest the anxiety itself. As outlined by the DSM, social anxiety sufferers may indeed fear that they “will act or appear in a certain way or show anxiety symptoms, such as blushing, trembling, sweating, stumbling over one’s words, or staring, that will be negatively evaluated by others […]” (APA, 2022: 230).

Alterations of bodily experience are also at the core of some phenomenological accounts of SAD, where attention has been drawn to the presence of disruptions of bodily self-consciousness (cf. Tanaka, 2021; Bortolan, 2022). In order to introduce these views below, I will first briefly outline some of the conceptual distinctions that have informed the broader phenomenological literature on the topic.

Phenomenological explorations of the body have distinguished between various forms of bodily consciousness, emphasising the primacy of the *lived body * (Husserl, 1989; Merleau-Ponty, 2012), namely a “first-person” experience of the body, or an experience of the body “as subject”(cf. Gallagher & Zahavi, 2012: 154 ff.). They have suggested that, typically, we do not take the body as an object of attention or observation. When we are immersed in our everyday activities, we do not think about the body, or monitor it; rather, we tend to be focused on our tasks, and objects and people in the external world. Despite this, we are still aware that we have a body, of its location, and general conditions. For example, we are able to immediately identify the position of our limbs and to perform a range of movements without monitoring them, and we typically have an immediate sense of our bodily capacities and potentialities (Gallagher and Zahavi, 2012: 155; Legrand, 2007: 499).

Drawing on this, research in classical and contemporary phenomenology suggests that we have a “pre-reflective” consciousness of our body: we have an awareness of the body and some of its features prior to reflecting on them (cf. Gallagher & Zahavi, 2012: 163 ff.). This has led some authors to observe that the lived body is often “transparent” (e.g. ibid.; Legrand, 2007). This means that the body is not something that is very noticeable or salient for us. Rather, we ‘see’ through it, so to speak: the body is that through which we experience the world (Merleau-Ponty, 2012: 94), and it is in the background or at the periphery of our experiential field.

However, pre-reflective consciousness is not the only way in which we can be conscious of our body. At times, the body becomes for us an object of attention and observation (cf. Gallagher and Zahavi, 2012: 164 ff.): in these cases, we are conscious of the body in a similar way to the way in which we are conscious of other objects. This is, for instance, what happens when we inspect our body, monitor its activities, or simply think about it.

In addition, we are reflectively aware of our body when we are trying to see it from the perspective of another person. For example, when we imagine how we would look or sound to an observer at a particular time. This amounts to being conscious of one’s body as an object of attention or observation *for another*, and it has been argued that this is what may happen when emotions like shame are experienced (cf. Sartre, 1989; Fuchs, 2002).

Phenomenological research on social anxiety has suggested that alterations of reflective bodily consciousness are also central to the experience of those who suffer from social phobia. For example, Tanaka (2021) has suggested that in social anxiety the experience of the body “as-object for others” has specific characteristics, and, in particular, it is accompanied by an increased sense of uncertainty or danger, due to the “unknowable” character of other people’s mental states.

In addition to this, it seems that social anxiety is marked more generally by a heightening of reflective self-consciousness, and, in particular, reflective bodily consciousness. Here the body is more often in the foreground (rather than in the background) of one’s experiential field, with the subject being acutely and persistently aware of how it may be perceived and judged by others (Bortolan, 2022).^4^

A study by Hackmann and colleagues (1998: 9–10), for example, found that those suffering from social phobia were more likely to experience “spontaneously occurring images when anxious in social situations”, and that these “were markedly from an observer perspective”, revolving around the way in which the subjects would appear to others. For instance, Hackmann and colleagues report that a patient worried about blushing, described her image as follows:

It’s like a camera zooming in on a horrible, red, panicky face, just the face and neck and top part of the body. I look really put-on-the-spot and nervous. (1998: 9)

Another patient offered the following description of how she was picturing herself during an episode of social anxiety:

Picture of me looking guilty, nervous, anxious, embarrassed. It’s my face—features distorted, intensified, big nose, weak chin, big ears, red face. Slightly awkward body posture, introverted body posture, turning in on myself. Accent more pronounced. I sound stupid, not articulate or communicating well. (1998: 9)

So, while we all become aware of our bodies from time to time, in social anxiety self-consciousness can acquire particular prominence, and its reflective forms are exacerbated. This can negatively affect personal and social agency, as having oneself at the forefront of one’s attention can make it very difficult to act promptly and spontaneously in response to other people’s inputs in interaction (Bortolan, 2022: 317; Beidel and Turner, 2007: 30).

## Being socially anxious on the internet

The pandemic of Covid-19 and societal responses to it triggered for various people a transformation of bodily experience, and such transformation often involved intense forms of anxiety. Under the threat of Coronavirus disease, both our own and other people’s bodies became objects of attention and scrutiny as potential vectors of infection and in their vulnerability to illness, and this has often caused fear and intense worrying (cf. Dolezal, 2020; Trigg, 2022). In addition, many found the shift to online communication difficult, and have reported that relating to others via internet-enabled technology was tiring and draining, making them hyper-conscious and less spontaneous.

However, as previously mentioned, these experiences were not universally shared, and for some anxiety sufferers, for example, the move online of social and professional activities was perceived as having a positive effect on mental wellbeing (cf. Cantor, 2021; Jarral, 2020; Khazan, 2021). In this section, I want to explore how the shift to online interaction may have affected the experience of social anxiety, unearthing the dynamics that may have generated these transformations.^5^

As outlined previously, the phenomenology of social anxiety often involves an increased consciousness of oneself as an object of attention and observation for others. Integral to this may be the sense that certain bodily parts or features are particularly visible or noticeable, and certain traits, characteristics or reactions are perceived as very salient or conspicuous: one’s tone of voice is not quite right; the blushing on one’s cheeks too intense; one’s movements too cumbersome. Social anxiety sufferers may feel continuously exposed to the scrutinising gaze of others, and the body is experienced as a catalyst for negative evaluations.

These dynamics may be partially offset when interacting online due the reduced level of exposure of the body in communication and interaction. In internet-mediated activity one’s body can be visible to others, for example in the case of video calling; however, this involves very different conditions from the ones we experience in encounters offline.

First of all, the portion of one’s body that can be seen by others at any given time is smaller than it is often the case when we speak to others in person. Typically, on a video call, only one’s face or the upper part of the body is caught by the camera, and we can choose what is captured in the relevant shot. As such, while we know that certain body parts are visible to others, we also know that other parts are hidden from sight, and hence there is a smaller risk that they will be noticed or judged by an observer.

In addition, due also to what have become acceptable practices online, we do have a higher degree of control over our visibility when taking part in a video call. Generally, on the relevant platforms, participants have the possibility to turn on and off their video streaming at any point, and it is not uncommon to do so during professional or social gatherings. As such, while in these circumstances we may still feel exposed to the gaze of others, this can be a much more intermittent experience, due to our own or other people’s choices. Furthermore, in this context exposure can have a more selective character: I can be heard without being seen or vice versa, and I can also be neither heard nor seen, should I or others want this to be the case. As highlighted by Osler (2021), on the internet, the experience of the body ‘as an object’ can be diminished, and my suggestion is that this can help social anxiety sufferers to reconfigure and improve their experiences in interaction.

It may be objected that this suggestion clashes with the fact that platforms like Zoom may intensify reflective self-consciousness by giving us the possibility to continuously monitor both ourselves and others through the images on screen. Some people indeed report that video calling makes them hyper-aware of themselves, and experiences like the so-called “Zoom fatigue” can be connected to this increased level of attention for the way in which we look or sound (cf. e.g. Bailenson, 2021).

I do not wish to deny that internet-mediated communication can result – for some people in some circumstances – in a heightening of self-consciousness. However, in this study I am exploring the reasons why this is not a universal experience, especially for those suffering from social anxiety. In this regard, it is certainly plausible that, for instance, the use of video calling platforms (especially when users are not very familiar with the medium) may exacerbate awareness of self and others and, potentially, anxiety itself. Nevertheless, it is important to recognise that these dynamics can also be offset by features of the media in question, for example the fact that, as mentioned above, online we can better control how visible ourselves and others are. So, here I am not claiming that every way of using these media is beneficial, but rather that certain uses have the potential to be.^6^ In addition, in doing so, my analysis also highlights how social anxiety sufferers may experience online and offline environments in a way that can be very different from (and sometimes opposite to) what is the case for those who are not socially anxious.

The account I am proposing is also consonant with empirical research concerning the experiences of social anxiety sufferers online. For example, a meta-analysis of research on the topic conducted by Prizant-Passal and colleagues (2016) points to the fact that those who are socially anxious tend to find online interaction more comfortable, and that there is a positive correlation between the smaller role played by non-verbal cues online and the experience of feelings of comfort in this space. The fact that anxiety signs, and one’s own appearance, can be concealed from others online – alongside a greater controllability of one’s experiences overall – have also been identified as key aspects of the improvements experienced by social anxiety sufferers on the internet (Erwin et al., 2004; Weidman et al., 2012).^7^

The features of online communication allow for exchanges that can be managed by the experiencer in a more direct way, and this makes it possible to handle more effectively the aspects of social interaction that are potentially distressing. This enhanced level of control also entails that we can act earlier than we would otherwise be able to do to mitigate the effects of a challenging situation. If I am feeling increasingly uncomfortable due to perceiving myself as the focus of other people’s attention, I can turn off the camera and/or the microphone for a while, so as to prevent myself from becoming engrossed in anxious feelings or overwhelmed. This is something that it would be more difficult to achieve in an in-person interaction, since, in order to disentangle ourselves from social situations, we are typically required to at least make the effort to abide by certain rules: we are expected to have specific reasons to interrupt an interaction, and to politely excuse ourselves for doing so. Getting outside of an interlocutor’s visual field is a much more effortful and codified endeavour when we are face-to-face than when we are online. Interactions on Zoom can be simpler and thus perceived as less threatening and easier to navigate. As explained by Olga Khazan (2021: para. 6) when discussing the experience of social anxiety :

Social anxiety is driven by a fear of being perceived negatively by others because you’ve misunderstood the subtle norms of a situation. But on Zoom, the rules are simpler. There’s no handshake, no decision about where in the room to sit, no need to even pick out an outfit from the waist down.

The more controllable character of communication on the internet has also to do with the fact that we can expose ourselves to it more gradually. A social anxiety sufferer entering a room to attend a meeting is immediately exposed to a range of events that could trigger and/or exacerbate the experience of being observed and being the object of negative evaluations. When interacting online, however, we can be much more deliberate about the rapidity and degree to which others can observe us and involve us directly in communication. For example, we are often able to enter meetings ‘discreetly’, appearing or disappearing from the screen without our entrance or exit being marked with greetings or remarks. Although practices may vary in this regard, it is indeed rare that the appearance or disappearance of new participants is explicitly noticed or commented upon, especially in the case of big gatherings. Contrary to what is the case when walking into a room full of people, online we can typically join without being heard or seen: with our microphone and/or camera off our presence can be signalled only by a name on the screen.

Another potential advantage of online communication and interaction concerns the possibility that these provide to be more intentional and careful in our expressions and responses to others. The pressure to react immediately to what others are saying or doing can be very high in face-to-face conversations, where the promptness with which a reaction is required can be communicated not just verbally, but also non-verbally through bodily posture, gestures, and movements. The way in which an interlocutor is sitting or gesticulating, for example, can make us feel under a great pressure to respond in a timely and appropriate manner. Research on the phenomenology and, in particular, embodied dimension of social interaction can further corroborate this point, as it shows that our responses tend to be in sync and attuned to the expressions, both bodily and emotional, of our interlocutors (e.g. Fuchs, 2013; Gallagher & Zahavi, 2012: 208 ff.).

These dynamics appear to be weakened, at least to an extent, when interacting online. Similarly to what is the case when speaking over the phone, people sometimes report that they have difficulties identifying when it is the right time to speak or respond, which may result in awkward moments, for example when two interlocutors start or stop talking exactly at the same time. “‘You go first’, ‘No, you go first’” situations seem to appear more frequently in the context of technology-mediated communication, and this may be due to the fact that here there are fewer bodily cues to guide our reactions. Indeed, we may not be able to see the other person’s face or body at all, and even when we do, their expressivity may be toned down by the medium or the lack of context. For example, it may be difficult to gauge whether someone really wants to continue a conversation if we cannot clearly discern their facial expressions on camera, or we do not see what their current environment is like.

So, online we may be less aware of other people’s bodily expressions, gestures, and movements, and, as a result, we may be less prone to respond to them in an immediate and emotionally consonant manner, thus exhibiting responses that are more pondered and intentionally crafted.

This is facilitated by the fact that also video calling platforms generally offer a chat option, giving users the possibility to communicate in writing in addition, or as an alternative, to speaking on a call. This is an aspect that has become well integrated in our practices during the pandemic, as sharing comments through the chat function – during Zoom calls, for example – has become a way of participating in meetings that can be no less valuable or sophisticated than discussing through a microphone.

The possibility of typing one’s points rather than conveying them orally may allow people to better prepare their interventions, as we are given more time to think and phrase our thoughts. This may have a particularly beneficial effect for those for whom speaking in front of others may engender uncomfortable feelings, as it may be the case in social anxiety. This dynamic is outlined, for instance, in the following remarks by Matthew Cantor (2021: para. 5), which are part of a broader discussion of the impact of working from home during the pandemic: 

[...] these days, most of my work-related interaction is through office chat apps. This means I have time to craft my comments before I utter them. Copy-editing often raises intricate queries that I struggled to stammer out in person; now, I can spend a minute writing them down as clearly as possible, making a better case for the changes I’d like to make. The slowed interaction gives me time to think, allows me to write considered arguments, and makes me more articulate – essentially, it allows me to be more of the person I aspire to be.

This appears to be in alignment with other reports concerning the degree to which communicating online during the pandemic has changed the dynamics of group meetings, for example by facilitating and broadening participation. As far as the experience of social anxiety is concerned, for example, Khazan (2021) observes the following:

[…] with Zoom, I’ve found myself feeling more relaxed, more emotionally regulated, and better able to advocate for myself. I feel as though I can more easily speak up in big meetings, and I can express myself to my bosses without worrying about my self-presentation. To me, Zoom turns everyone else into fake people—not people with power over me, just little faces in boxes on my screen.

Cantor’s and Khazan’s observations draw attention to how the lack or diminishment of face-to-face interaction in online communication may allow for more deliberate and assertive expressions. In addition, these reports point also to another feature of internet-mediated sociality, namely the fact that this can allow for a higher degree of authorship over one’s experience. The way of communicating that is afforded by the use of chats, Cantor (2021) observes, makes it easier “to be more of the person” that one wants to be, first of all because it gives more time to craft one’s actions and reactions.

Interacting online may enhance our capacity to shape our experiences also because it gives us the opportunity to better control how those experiences are narrated to ourselves and others. When exchanging messages in a chat, for instance, I can sound confident even if I am blushing, or I can say something courageous even if I am shaking, and this is the case, even if not always, during video communication online too. As discussed above, I can turn my camera off if I do not want my interlocutors to read embarrassment on my face, or I can at least carefully select how they see me. If confidence and courage are valuable features for me to achieve and display, this mode of communication makes it easier for me to appear – to others, but also to myself – as someone who embodies those traits.

This does not mean that online communication facilitates deception (although, in some cases, it may as well do). Rather, this example illustrates how on the internet it may be easier to enact features or behaviours that we have been trying to achieve, being less affected or discouraged by potential setbacks. For instance, when interacting in person, my willingness to behave confidently could be easily undermined by one ‘failure’ to display such behaviour: without the possibility of modulating our exposure to the other person, an involuntary blushing or a brief shaking of the voice may have a ‘ripple effect’, causing us to become hyper-aware of our bodily responses and less able to seamlessly continue the interaction. However, online, thanks to the flexibility we often have in regulating the visibility of our reactions, we could change the trajectory of our responses earlier on, so that, despite the initial blushing or shaking, we could still enact behaviours that are consonant with the confidence that we want to embody and display.

## Emotion regulation online

So far, I have discussed various dynamics in virtue of which the experience of anxiety, and, in particular, social anxiety, may be mitigated through online communication and interaction. These insights are important in order to better understand the impact that internet-enabled technology can have on those who feel persistently or acutely anxious; however, it is possible to wonder to what extent what I have illustrated here can shed light on affective experience more broadly. In other terms, could these insights be extended, to account also for how other affects may be impacted upon in cyberspace?

Krueger & Osler (2019) have drawn attention to the important role that the experiences we have on and through the internet can play in modulating our affective states. More specifically, they have argued that online we can craft and inhabit “self-styled” spaces that provide a variety of tools to regulate our emotions.

The notion of emotion regulation refers to a range of processes through which we influence the experience and expression of emotion. According to Gross, this comprises “conscious and unconscious strategies” through which the experiential, behavioural, and physiological components of an emotion can be modulated (Gross, 2001: 215; Gross, 2008: 711) . 

In their work on the topic, Krueger and Osler observe how research concerning these regulative practices has often taken an “intrapersonal approach”, focusing on the individual and giving insufficient consideration to the role of environmental processes (2019: 208–209). This is something that their work aims to rectify, drawing attention to how a range of material and social resources are involved in emotion regulation on the internet.

While I agree with the idea that interpersonal dynamics are central to the modulation of affects, here I want to explore further the subjective side of emotion regulation, seeking to illuminate how it may be enabled online by specific transformations of bodily experience. To do so, in the following I will first outline the emotion regulation strategies identified by Gross, and will then explain how they can be implemented by a subject in the anxiety examples discussed before, and in online communication and interaction more generally. Through this, I do not aim to claim that emotion regulation is always, or overall, easier online than it is offline; rather, I want to outline the features and dynamics that can facilitate and support regulatory processes in the context of online exchanges.^8^

Gross (2001) puts forward a “process model of emotion regulation”, which identifies different times and ways in which an emotion that is being undergone can be regulated. In particular, he distinguishes between modes of emotion regulation that are “antecedent focused” and those that are “response focused”: the former are adopted before an emotion has been fully aroused, while the latter are implemented “once an emotion is already under way” (215). Antecedent-focused strategies include *situation selection*, *situation modification*, *attentional deployment*, and *cognitive change*, while response focused strategies involve *response modulation* (Gross, 2001, 2008).

Situation selection refers to the way in which we can influence our emotions by determining the circumstances in which we find ourselves. For example, I may invite a group of friends to spend the evening at my place, rather than remaining alone and spending the night working.

Situation modification refers to the manner in which we can impact on the circumstances we are in to generate certain emotional effects. For instance, when my friends are around, I can influence the atmosphere of the gathering by selecting certain music, for example cheerful or relaxing tunes.

Attentional deployment designates the acts through which we direct our attention to aspects of the situation that we have chosen to focus on. In the case of the evening party sketched above, I may seek to follow closely the board game that we are playing, rather than listening to what a friend who has picked up a call is saying.

Cognitive change designates the processes through which we interpret what we are paying attention to in certain ways. An example of this is “reappraisal”, which “means that the individual reappraises or cognitively reevaluates a potentially emotion-eliciting situation in terms that decrease its emotional impact” (Gross, 2001: 216). To illustrate this further through the use of the example previously introduced, we can imagine that a friend of mine was unusually quiet through the evening, and I got worried that they did not enjoy my company. In this case, I can reappraise the situation by reading my friend’s behaviour as expressive of the fact that they were able to relax, rather than as a sign of boredom.

As mentioned above, response modulation refers to the strategies through which we may attempt to impact on the emotion after it’s been aroused, and one way of doing so discussed by Gross is “suppression”, which consists in attempting to inhibit the expression of the emotion (Gross, 2001: 216). Let’s imagine that a friend at the party has inadvertently poured a glass of wine on the carpet, and that I got annoyed at them for not being more careful. Here, suppressing the emotion may involve trying to keep a neutral facial expression or tone of voice when dealing with the accident.

It seems that all the emotion regulation strategies identified by Gross can be involved in the management of social anxiety during online communication and interaction, and that this could apply also to the experience and expression of other emotions on the internet.

Communicating and interacting in cyberspace often is a deliberate choice. I can decide to join and leave a Zoom meeting, to read or ignore a conversation on Whatsapp, to carefully check or just skim through my Facebook feed. As illustrated previously, in the case of social anxiety, this degree of control over the circumstances may enable me to prevent the arousal, or avoid the exacerbation of anxious feelings: if an online meeting or a conversation in the chat are becoming distressing, I can simply remove myself from the situation by leaving, which, as I highlighted before, can be done much more discreetly and seamlessly on the internet than in face-to-face interactions. These dynamics seem to be analogous to what described by Gross through the notion of “situation selection”, and it appears to be a feature of much of our activity online. In cyberspace we continuously choose ‘where’ to go, how long to remain there, and with whom we want to communicate, and these decisions have a significant impact on our ability to elicit and manage our emotions. For example, if we are looking to trigger or maintain a cheerful and light-hearted mood, opening social media where we know we will encounter friends may be a good choice.

It could be objected that interacting on the internet can also be an experience marked by instability and unpredictability, especially in the case of social media, where we may be exposed to a high number of changing stimuli and people. In virtue of this, it has indeed been argued that emotions online can be particularly intense (Ben Ze’ev, 2004), and this factor, alongside other features of online communication – such as the more overt expression of negative emotions like anger (Derks et al., 2008) – may suggest that emotion dysregulation can be fostered on the internet.

It is certainly important to take into account these dynamics when we explore the affective impact of online communication. However, it should also be noted that some of the destabilising events that can hinder regulation may be more common on certain platforms. As such, when we decide to communicate on the internet, we can give consideration to which technologies and settings might best allow us to select the stimuli to which we are exposed (e.g. making one’s posts visible only to a specific set of contacts), and act accordingly.

In addition, online communication and interaction also give us plenty of opportunities to modify the situations in which we find ourselves, in particular due to having a degree of control over when and how we appear to others. As discussed above, this can be very positive for those who suffer from social anxiety, in so far as it makes it possible to reduce the visibility of one’s body at times at which the anxious feelings may be arising or intensifying. For example, if I am becoming increasingly preoccupied with my interlocutor’s perception and evaluation of the way I look, I can turn off the camera, or change the perspective through which I appear. This, I believe, parallels what Gross describes under the label of “situation modification”, and can be seen too as a rather common way for us to manipulate our affects on the internet. For instance, we sometimes have the possibility to choose how the spaces we are in look to us and others: on various video conferencing systems, we can tweak our background, choosing from a range of pre-set options, or using our own photos. Similarly, on social media like Facebook or Twitter, we have control over a range of permanent features of the spaces that are visible to ourselves and others: every user on these platforms has a profile picture that remains constantly present somewhere on the screen, but the picture can be changed, and this is the case also for other ‘background’ features, like the cover photo positioned at the top of one’s personal wall or page. As such, while we cannot change the fact that on these networks we appear through a photo icon, and our individual pages include the space for a large cover picture, we can effectively ‘furnish’ these spaces as we want to generate or favour certain emotional experiences.

Attentional deployment is also a form of emotion regulation that seems to be central to both the management of anxiety and other affects on the internet. For example, on video conferencing systems we may have the option to choose how to visualise other participants: on Zoom, we can choose if we want to see everyone on screen in the same way (the ‘gallery’ view) or whether we want the person who is speaking to be visible as a bigger icon at the centre of the screen (the ‘speaker’ view). In addition, where a chat function is available, we typically can decide whether to look at it or not (although we may be automatically notified when there are some unread messages). Paired with the control we have on the way in which we appear to others on these media, it seems that these mechanisms may enable us to more effectively direct or redirect our attention when needed. For instance, if I am giving a presentation on a video conferencing platform, and I become concerned with what the facial expressions of my audience may signal about the perceived quality of my performance, I may select a view that reduces the visibility of members of the audience, thus mitigating the feeling of being observed from numerous perspectives. Furthermore, on social media, we may be able to exercise some control over which information we receive, for example, selecting to prioritise certain updates on a Facebook feed. This seems an important way of managing our attention by making it easier to focus on stimuli that are consonant with certain emotions, while ignoring others.

There are various reasons to think that interacting on the internet can also facilitate cognitive reappraisal. This may occur, for instance, due to the different timeframes within which we might be communicating online. Having a conversation in a chat, for example, allows for different response times from those that are expected in face-to-face conversations. Even when chatting synchronously, it is indeed common, and considered acceptable, that one may not reply to a message immediately. This allows the parties in the conversation to potentially take more time to ponder over their responses and the responses of their interlocutors, making it possible to appraise the situation in different ways. This may be a helpful dynamic for those who experience social anxiety, providing the opportunity to re-assess one’s take on a situation before further progressing in the interaction. For example, if I am exchanging messages with a friend, and their responses are becoming shorter, I may start thinking that this is the case because what I am saying is boring and I can’t make conversations interesting. However, the lack of pressure to reply immediately gives me the chance to challenge this initial evaluation, considering alternative possibilities that may reduce my anxiety: perhaps my friend’s kids are asking for their attention, or they simply do not have much to add to the particular points we are discussing.

Finally, response modulation too appears to be a way of regulating one’s emotions that can be rendered easier when interacting online. As I outlined before, one of the peculiarities of communication on the internet is the increased degree of control that it can give over the way in which one appears, and on one’s visibility more broadly. This means that emotional expressions can be more easily hidden or camouflaged if one does not want others to be aware of how they are feeling.

With regard to this point, however, it may be objected that making one’s emotions invisible or difficult to decipher on the part of others is not the same thing as suppressing their expression: for example, one may still be blushing due to feeling embarrassed, or one may still cry due to being sad, thus making it the case that the relevant responses are not really being modulated. While this seems a plausible point, it is nevertheless worth noting that controlling the visibility and appearance of one’s own reactions can have a significant impact on the way in which others respond to them. For example, if others do not realise that we are emoted in a certain way, they will not respond in ways that will require us to acknowledge the existence of the emotion: for instance, they will not ask if something is troubling us, or if we want to take a break or interrupt the meeting we are in. The lack of response on the part of others, in other terms, makes it possible for us to not have to dwell on the emotion itself, which in turn can facilitate its suppression.

## The possibility of healing online and its ethical dimension

So far, I have argued that, while for many this has been distressing and difficult to navigate, the shift to online communication and interaction occasioned by the pandemic has also engendered positive changes for some people with a lived experience of mental ill-health. In particular, I have argued that the different manner in which bodies can be perceived and related to online can mitigate social anxiety, and, more broadly, can facilitate emotion regulation.

My analysis is consonant with the idea that the way in which the body is involved in intersubjective and social experience in cyberspace is rather different from what is the case in offline interactions. More specifically, I agree that “intercorporeality”, namely the range of interpersonal expressive and affective exchanges that are mediated by the body, is lessened when we are in cyberspace (Dolezal, 2020). However, I do not think that this form of ‘dis-embodiment’ is bound to have a negative impact on self- and other-experience (although it can have such an impact in some circumstances).

Through this study, I indeed have shown that the ways in which we communicate and interact with each other online can also help to re-configure our affective experiences in ways that are potentially transformative, having a positive impact on some forms of mental ill-health.

While I have suggested that this is favoured by the diminished centrality of the body in cyberspace, I do not share the view that online we cannot be with each other in ways that are as meaningful and fulfilling as offline. Phenomenological research on this topic has highlighted that interpersonal understanding, emotional sharing, and we-experiences can indeed occur via internet-mediated technology (e.g. Osler, 2021; Krueger & Osler, 2019). More broadly, Osler and Krueger (2021) have shown that we can inhabit and construct online spaces that afford forms of relatedness that are not necessarily weaker or worse than those supported by face-to-face environments. On the contrary, they argue that “interaffectivity” and “expressivity” can still permeate the way in which we relate to each other on the internet, thus making it the case that the social worlds developed via these means are not always impoverished (although they may have distinct pitfalls and may generate specific challenges).

This study supports the idea that embodiment, affectivity, and sociality are malleable dimensions, and that there are good reasons to continuously explore the diverse configurations of experience in which such dimensions may manifest. As highlighted by Petherbridge (2022), the pandemic has led us to radically change some of our bodily and social habits, a transformation which has had ethical significance, in so far as it was motivated by the need to protect ourselves and others from the virus. However, the transformation of our habitual ways of relating to each other has also brought to light how different forms of communication and interaction are possible, and how, in some circumstances, they can support improvements to wellbeing.

This, I believe, has implications for the way in which we conceive and plan our lives in ‘post-pandemic’ times, and, in particular, it challenges the idea that a return to ‘normality’ should amount to a complete return to the practices that were predominant prior to Covid-19.

Research concerning the impact that the pandemic has had on mental health has shown that the measures which have been implemented to counteract the spread of the virus have led to alterations of various dimensions of experience, which also appears to have exacerbated some pre-existing psychopathological disturbances (Lau et al., 2022). While this is significant data to be taken into account, it is also important to consider that the changes engendered by the pandemic were of different kinds, even if they were interrelated (e.g. the shift to remote working occurred during lockdowns, but could also take place when societies are completely ‘open’). As such, it is possible that, outside of a pandemic, some of the changes that became widespread due to Covid-19 could have had different effects. In addition, in line with the insights advanced in this study, empirical research has also shown that some people with an experience of mental illness have not suffered a worsening of their condition during Covid-19, and rather have perceived some improvements (see e.g. Thygesen et al., 2021).

The analysis I have developed in this study aimed to bring to light some of the positive experiences that, among others, some anxiety sufferers had during the pandemic, suggesting that their potential to foster wellbeing is dependent on factors – i.e. their capacity to support emotion regulation – that could be leveraged in the post-pandemic world.

Reverting to the habits around which most of our lives were structured before 2020 – as suggested by those who advocate for the swiftest replacement of online or hybrid activities with in-person events – may thus run the risk of obliterating the experiences and possibilities that some people have been able to benefit from as a consequence of the pandemic. I suggest that these experiences and possibilities should be explored and drawn upon as we progress to shape and re-shape our life in the aftermath of Coronavirus, as in so doing we might be able to think of more inclusive, diverse, and health-enhancing ways of structuring our personal, social, and professional lives.

## Footnotes

1 Phenomenological research in this area has also contributed to highlight how experiences of social distancing have not been the same for everyone, and in particular, how, due to pre-existing conditions of inequality, disadvantage, or discrimination, some social groups have been affected more negatively than others by the measures adopted during the pandemic (Dolezal & Lucas, 2022).

2 The extent to which experiences and emotions on the internet are “disembodied” can be debated (cf. Benski and Fisher, 2014: 2 ff.; Osler, 2021). In this study, I use the notion of “disembodiment” to refer to the diminished centrality of certain forms of bodily experience in online communication and interaction.

3 The term “social phobia” has also been used in some editions of diagnostic manuals (e.g. WHO, 1992) to refer to “social anxiety”. In this paper I use the terms interchangeably.

4 I believe that there is a degree of continuity between ordinary and pathological forms of anxiety, in so far as they involve experiences with analogous structures, and in the rest of this study I focus on features of anxiety that can be more or less intense or disruptive but are not necessarily associated with a condition that could be diagnosed as a disorder. However, some anxiety experiences can be very distressing and/or debilitating enough to significantly interfere with the person’s life, and these instances may be captured by what has been referred to as SAD and social phobia.

5 Research conducted prior to the pandemic had shown that those who suffer from social anxiety tend to use the internet more than those who are not socially anxious for social interaction, and to mitigate social concerns (cf. Shepherd & Edelmann, 2005). The insights I develop in this paper – while motivated by the transformations brought about by Covid-19 – are relevant also to understanding internet use by social anxiety sufferers prior to this period.

6 In his exploration of the causes of Zoom fatigue, Bailenson (2021) suggests that on Zoom people tend to pay more attention than usual to non-verbal cues. As argued above, while I do not deny that this may be the case for many, in this study I am focusing on uses of these media that appear to have an opposite effect. In this regard, Bailenson also suggests that some of the causes of Zoom fatigue could be solved by changes to the software interface or to the uses that are made of it. As he explains: “[…] the default setting should be hiding the self-window instead of showing it, or at least hiding it automatically after a few seconds once users know they are framed properly. Likewise, there can simply be a limit to how large Zoom displays any given head […]. Outside of software, people can also solve the problems outlined above with changes in hardware and culture. Use an external webcam and external keyboard that allows more flexibility and control over various seating arrangements. Make ‘audio only’ Zoom meetings the default, or better yet, insist on taking some calls via telephone […]”(2021: Conclusion section, para. 2).

7 It could be noted though, that the higher level of comfort experienced by social anxiety sufferers online could also become an incentive to avoid the face-to-face interactions that have the potential to generate anxious feelings (cf. Prizant-Passal et al., 2016). Avoidance behaviours may provide temporary relief from anxiety, but they could hinder the capacity to regulate such feelings and potentially increase anxiety in the relevant situations. This, however, is not in tension with the insights developed in this study, in so far as here I am not suggesting that it would be appropriate to completely replace face-to-face interaction with internet-mediated one. Rather, I point out that the latter can have some benefits for social anxiety sufferers. For example, by being relieved from anxiety, people can more easily draw upon a wider range of affective and cognitive experiences, which can be helpful to manage the condition and can support longer-term transformative processes.

8 It is also worth noting that not all the regulation strategies discussed here may be viable or equally beneficial for every internet user at any time. For example, low levels of familiarity with internet technology may make it more difficult to incorporate this technology in one’s interactive practices and may be itself a source of anxiety.
